# Effects of Cerium on the Mechanical Properties of Al–20Si Alloy

**DOI:** 10.3390/ma19132707

**Published:** 2026-06-24

**Authors:** Liang Hu, Xiaoming Du, Haicheng Liang, Guanglin Zhu, Wenqi Han, Fengling Zhang, Junzhe Liu, Yiqiao Tang, Shuai Wang

**Affiliations:** 1 School of Materials Science and Engineering, Shenyang Ligong University, Shenyang 110159, China; huliang@sylu.edu.cn (L.H.); 13644298215@163.com (J.L.);; 2 School of Equipment Engineering, Shenyang Ligong University, Shenyang 110159, China; 3 School of Metallurgy, Northeastern University, Shenyang 110819, China

**Keywords:** hypereutectic Al–20Si alloys, chemical modification, rare earth cerium, mechanical properties, fracture mechanism

## Abstract

The chemical modification of Al–20Si alloy in the case of incorporation of rare earth cerium (Ce) is investigated in this study, focusing on the resulting microstructure evolution and changes in mechanical properties and hardness before and after cerium modification. The microstructure of the cerium-modified alloy is composed of the aluminum matrix, eutectic silicon, and primary silicon phases, as well as the Al_2_Si_2_Ce phase, a cerium-containing solid solution, and the α-Al_11_Ce_3_ phase. It is worth noting that this study reports for the first time an unusual phenomenon in which the addition of cerium raises tensile strength of the alloy while concurrently decreasing its surface hardness. The increase in tensile strength is mainly ascribed to the refinement of primary silicon. On the contrary, the reduction of hardness is explained by the competition between Ce-induced toughening and solid solution strengthening, as well as the generation of the relatively soft α-Al_11_Ce_3_ phase. Mechanical properties at room temperature show that the greatest tensile strength of 153 ± 7 MPa is reached at a cerium concentration of 0.7 wt.%, while the corresponding elongation of the mechanical specimen is 1.36 ± 0.287. The fracture mechanism changes from cleavage fracture in the unmodified alloy to ductile fracture after Ce addition.

## 1. Introduction

Aluminum-silicon (Al–Si) alloys are a widely used class of aluminum alloys, significantly employed in industries such as automotive, aerospace, and defense because of their lightweight nature, low thermal expansion, high specific strength, and excellent abrasion resistance [1,2]. Particularly, hypereutectic Al–Si alloys show superior mechanical properties with the increase in silicon content. Godbole et al. [3] identified hypereutectic aluminum–silicon alloys as promising materials for electronic packaging applications. These alloys offer a potential replacement for second-generation Fe–Ni–Cu alloys with controlled thermal expansion, achieving a weight reduction of approximately 30%. Their coefficient of thermal expansion (CTE), ranging from 7 to 17 × 10^−6^ K^−1^, closely aligns with that of ceramic substrates and semiconductor materials, rendering them particularly suitable for electronic packaging purposes. Liu and Chen [4] investigated the effects of Al–Ti–B and Al–Sr chemical modifiers on an Al–16Si–4Cu–0.5Mg–0.2Mn alloy. Their findings demonstrated that the modifiers significantly refined both the primary and eutectic silicon phases, which led to an enhancement in tensile strength by 65 MPa and an increase in elongation by 0.4% relative to the unmodified alloy. These improvements suggest the alloy’s applicability in automotive piston manufacturing. Furthermore, Lewis et al. [5] reported that hypereutectic aluminum–silicon alloys possess adjustable thermal expansion coefficients and exhibit high thermal conductivity, making them well-suited for structural heat sink applications. When combined with copper components, these alloys effectively mitigate deformation arising from mismatched thermal expansion coefficients, thereby confirming their high reliability under thermal cycling conditions. However, under conventional solidification conditions, these alloys tend to generate coarse primary silicon phases with complex morphologies, such as star-like, plate-like, stripe-like, and irregular shapes. The sharp edges of these large primary silicon particles cause the generation of stress concentration, leading to cracking within the aluminum matrix and ultimately degrading the mechanical performance of the alloy. Therefore, the control of the morphology for primary silicon has become a critical focus in alloy development research.

Many techniques have been published to optimize the primary silicon phase, including chemical modification [6,7], high-pressure solidification [8,9], rapid solidification [10,11], semi-solid stirring [2,12], directional solidification [13,14], and melt superheating [15,16]. Among these, chemical modification is particularly advantageous for large-scale melt processing techniques, such as casting and continuous casting, because of its operational simplicity, cost-effectiveness, and compatibility with complementary refinement strategies such as melt superheating and rapid solidification. A series of chemical modifiers, involving Al–Si–Fe [6,17], rare earth (RE) elements [18,19], nanodiamonds [7], Cu–P [20], Al_2_O_3_ [6], Sr [6,21], Mg [22], and Bi [23], have proven effective in refining primary silicon and significantly enhancing the mechanical properties of Al–Si alloys. It is important to note that RE elements are able to retain their refinement ability across multiple remelting and casting cycles, which achieves optimal refinement with minimal additions (typically less than 1 wt.%) and leads to alloys with superior mechanical property. Given these attributes, RE elements have been metaphorically referred to as the “vitamins of modern industry,” which attracts significant global research interest. Their applications extend beyond aluminum alloys to include magnesium and titanium alloys, which highlights their wide application in materials modification [24,25,26].

Chen et al. [27] produced a hypereutectic Al–25Si alloy by the means of rapid solidification and studied the effects of four distinct modifiers: La, Ce, La–Ce, and La–Ce–Y. Their findings indicate that cerium had a more significant refining effect on primary silicon relative to lanthanum, although both effectively refined eutectic silicon. The use of dual or triple RE modifiers (La–Ce and La–Ce–Y) gave rise to significant refinement of both the primary and eutectic silicon relative to single RE additions. It is worth noting that the La–Ce–Y combination provided the most significant refinement of primary silicon because of yttrium’s strong adsorption affinity for the primary silicon surface, which inhibited its growth and generated fine plate-like morphologies. Consequently, the alloy reached an optimal room-temperature tensile strength of 150 MPa, a 33.9% improvement over the unmodified alloy’s 112 MPa. Mao et al. [19] studied the effects of dysprosium on hypereutectic Al–Si alloys and discovered that dysprosium effectively refined both primary and eutectic silicon at an optimal concentration of 0.15 wt.%. The morphology of primary silicon transformed from irregular polyhedrons and dendrites to fine polyhedral particles, with the average size decreasing from 48 to 38 μm. The eutectic silicon transformed from coarse, uneven short rods into a fibrous structure. The modified alloy indicated a tensile strength of 233 MPa. In addition to aluminum, silicon, and dysprosium, the alloy contained trace amounts of iron, copper, manganese, titanium, and phosphorus, which facilitated subsequent heat treatment processes. After T6 heat treatment, fragmentation of the as-cast Al–Si–Fe–Mn and Al_2_Cu phases further increased the tensile strength to 242 MPa.

Jing et al. [18] used a combined approach of lanthanum (La) modification and molten salt purification to modify the microstructure of an Al–20Si alloy. They began by melting a mixture of KCl, NaCl, and Na_3_AlF_6_ in a magnesia crucible at 850 °C. The molten salt was then immersed into the Al–20Si melt for purification. Next, they added an Al–10La master alloy into the purified melt for modification. The La-modified alloy was then cast into a mold. Their results indicated that La partially prevented the nucleation of primary silicon in hypereutectic Al–Si alloys. The combined application of 0.3 wt.% La and molten salt purification fully suppressed the generation of primary silicon, which leads to a pseudo-eutectic microstructure. The mechanical properties at room temperature showed that the modified alloy reached a tensile strength of 181 MPa, representing a 19.7% improvement relative to the unmodified alloy. These findings highlight the synergistic effect of La addition and molten salt purification in controlling solidification microstructures and enhancing mechanical performance. Chen et al. [28] investigated the combined modification of the Al–20Si alloy using phosphorus (P) and RE elements. Phosphorus primarily refined primary silicon by reacting with aluminum to form aluminum phosphide (AlP). Because both AlP and silicon have diamond cubic crystal structures with closely matched lattice parameters (Si: 0.542 nm; AlP: 0.545 nm), AlP particles served as heterogeneous nucleation sites for primary silicon during solidification, leading to significant refinement. Moreover, RE elements helped in the refinement of both primary and eutectic silicon. For eutectic silicon, RE atoms preferentially adsorbed onto growth steps, twin boundaries, and Al–Si interfaces, which lowered the eutectic temperature and inhibited silicon growth. In the case of primary silicon, grain refinement resulted from a decrease in nucleation temperature and changes in the solid–liquid interface energy and silicon surface energy. The combined effect of P and RE reduced the average size of primary silicon from 66.4 μm to 23.3 μm and eutectic silicon from 8.3 μm to 5.2 μm. Consequently, the tensile strength was raised from 256 MPa to 306 MPa. The incorporation of rare earth elements—including Er [29], La [18], Nd [30], Y [31], Sm [32], Yb [33], Eu [34], and Ce—into hypereutectic aluminum–silicon alloys markedly enhances both tensile strength and elongation, as shown in Table 1. While neodymium (Nd) yields the most significant improvement in tensile strength, lanthanum (La) and cerium (Ce) are more favorable for industrial applications due to their comparatively lower costs. Specifically, the price of La is $3007.35 per ton, and that of Ce is $5097.82 per ton, whereas the prices of other rare earth elements exceed $11,002.50 per ton.

Previous studies have shown that RE elements are able to effectively control the morphology of silicon phases, either individually or in combination with other modifying agents, thereby increasing the mechanical property of alloys. Nevertheless, the present investigation uncovered an unusual phenomenon: the addition of cerium (Ce) improved the mechanical properties of the alloy while concurrently reducing its hardness. Thus, tensile strength and hardness showed an inverse relationship, which contradicts earlier studies reporting a positive correlation for elements such as magnesium (Mg) and phosphorus (P) [35,36]. This atypical behavior of RE elements is scarcely reported in the existing literature. In general, hardness correlates positively with strength parameters, including yield and tensile strength, as it reflects a material’s resistance to localized plastic deformation, a property intrinsically related to dislocation mobility. Materials of higher strength generally indicate greater resistance to dislocation movement. Hence, this study focused on the Al–20Si alloys to investigate the effects of Ce on microstructure evolution, particularly with the purpose of clarifying the mechanisms behind the observed negative correlation between mechanical properties and hardness in the existence of Ce.

## 2. Experimental Details

### 2.1. Material Preparation

The aluminum pellets (6-mm diameter, 99.999 wt.% purity), Al–10Ce master alloys, and silicon blocks (grade 1101#, 99.9 wt.% purity) were used in this study. Four distinct alloy compositions were prepared by means of conventional casting techniques. The nominal chemical compositions of these alloys is enumerated in Table 2. The alloy melting procedure involved placing silicon and aluminum pellets in a graphite–clay crucible, which was preheated to 300 °C for 30 min to get rid of moisture. The temperature was then raised to 750 °C and maintained for 30 min to ensure full dissolution of the silicon. Following this, the temperature was increased to 800 °C, at which point the Al–10Ce master alloy was added to the melt. The molten mixture was maintained for 10 min to promote uniform distribution of the cerium, followed by degassing using a refining agent and slag removal. Finally, the molten alloy was cast into a 304L stainless steel mold at 760 °C, which leads to cylindrical ingots with diameters of 15 mm.

### 2.2. Metallographic Specimen Preparation

Wire electrical discharge machining (WEDM) was used to cut the as-cast ingots into cylindrical specimens measuring 12 mm in diameter and 10 mm in height. These specimens were cold-mounted and subjected to sequential grinding with abrasive papers of grit sizes 80#, 240#, 600#, 800#, and 1500#. The polishing process was completed using diamond grinding pastes of 2.5 and 1.0 μm particle sizes to achieve a mirror-finish surface, thereby finalizing the preparation of the metallographic specimens.

### 2.3. Mechanical Specimen Preparation

**Figure 1:**
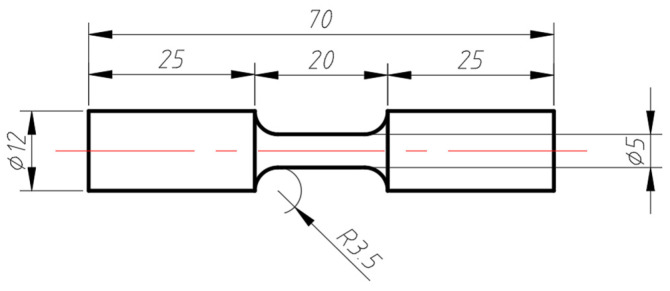
Schematic detailing the dimensional specifications of the mechanical tensile test specimen fabricated from the Al–20Si–Ce alloy [37].

Tensile specimens were fabricated by WEDM according to the dimensional specifications outlined in the mechanical testing schematic (Figure 1 [37]).

### 2.4. Performance Characterization

Microstructural modifications of the alloy were analyzed using a GeminSEM 300 field-emission scanning electron microscope (Carl Zeiss AG, Oberkochen, Germany) equipped with an energy-dispersive spectroscopy (EDS) detector to determine elemental composition. In this study, Digital Micrograph 3.0 software (v2021) was employed to perform a statistical analysis of the grain sizes of thirty primary silicon grains observed on the alloy surface under the SEM before and after cerium modification. Phase identification was performed via X-ray diffraction (XRD) analysis using an Ultima IV diffractometer (Rigaku Corporation, Tokyo, Japan) with Cu-Kα radiation (λ = 1.5406 Å) and a graphite monochromator. The XRD measurements were conducted under the following conditions: an accelerating voltage of 40 kV, a current of 40 mA, a step size of 0.05°, and a 2θ scanning range from 20 to 100°, at a scanning rate of 10° per minute. Further microstructural investigation was conducted using a high-resolution transmission electron microscope (FEI Talos F200S, Thermo Fisher Scientific, Waltham, MA, USA) to observe the fine structural details in the alloy. An AGV-V computer-controlled universal testing machine (Shimadzu Corporation, Tokyo, Japan) was employed to assess the mechanical tensile properties at ambient temperature, with a constant tensile rate of 1 mm/min applied to determine the ultimate tensile strength (UTS), yield strength and elongation. For each alloy composition, three mechanical test specimens were prepared.

Microhardness measurements were performed on the primary silicon phase, Al–Si eutectic, and aluminum matrix using a microhardness tester (FM-300 model; Future-Tech Co., Ltd., Izumi City, Japan). The conditions for the microhardness test are specified as follows: a test load of 1000 gf is applied, with a dwell time of 45 s. For each phase, five independent measurements were conducted and averaged to obtain statistical reliability.

## 3. Results and Discussion

### 3.1. SEM Microstructural Characterization of the Unmodified Al–20Si Alloy

**Figure 2:**
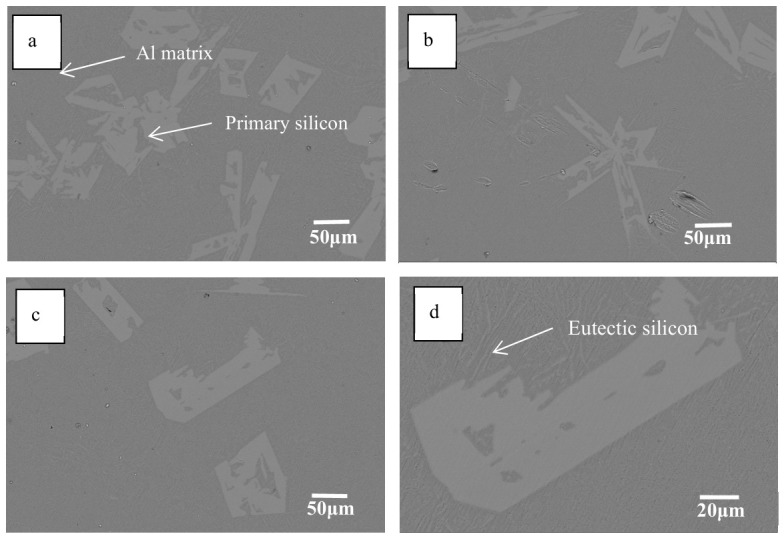
SEM images of the microstructure for the Al–20Si alloy: (**a**) irregular primary silicon morphology; (**b**) primary silicon indicating a five-petal star-shaped morphology; (**c**) lamellar primary silicon morphology; and (**d**) morphology of the eutectic silicon and primary silicon.

Figure 2 exhibits SEM images of the microstructure for the Al–20Si alloy. It can be seen from Figure 2a–d that the unmodified alloy is mainly composed of an aluminum matrix, Al–Si eutectic phases, and primary silicon phases. The primary silicon shows a series of complex morphologies, which includes star-like, plate-like, and irregular shapes. This complexity of morphologies is ascribed to several factors related to crystal growth conditions, alloy composition, and cooling rate, detailed as follows.

(1) Crystallographic anisotropy: The growth rate along the Si<111> crystallographic direction is significantly greater than in other orientations, which results in the preferential growth of primary silicon and the generation of plate-like, polyhedral, or star-shaped structures. This anisotropic growth behavior is particularly significant under non-equilibrium solidification conditions. Branching or tip splitting may occur during the process of growth because of these anisotropic effects, which further contributes to the observed irregular morphology.
(2) Effects of cooling rate: Under slow cooling conditions approaching equilibrium, silicon atoms get enough time to diffuse to the crystal surfaces, which promotes the generation of coarse plate-like or polygonal primary silicon structures. In comparison, rapid non-equilibrium solidification limits diffusion of silicon atoms, which leads to finer blocky, fibrous, or honeycomb morphologies, thereby increasing the complexity of the primary silicon microstructure.
(3) Interaction with alloying elements: The existence of modifying elements such as phosphorus (P), strontium (Sr), or sodium (Na) in the melt can give rise to their adsorption on silicon crystal surfaces, which in turn changes the growth kinetics of the primary silicon. For instance, phosphorus can react with aluminum to generate AlP compounds. These compounds are as heterogeneous nucleation sites, which promotes the refinement of the primary silicon phase [6].

To sum up, the above factors contribute to the complex morphology of the primary silicon in the unmodified Al–20Si alloy. Moreover, Figure 2d indicates needle-like eutectic silicon, indicative of eutectic solidification processes that produce Al–Si eutectic structures, where silicon presents as eutectic silicon. Chemical modification of primary silicon through appropriate modifiers has significant potential for increasing the mechanical properties of the alloy.

### 3.2. SEM Microstructural Analysis of Ce-Modified Al–20Si Alloy

**Figure 3:**
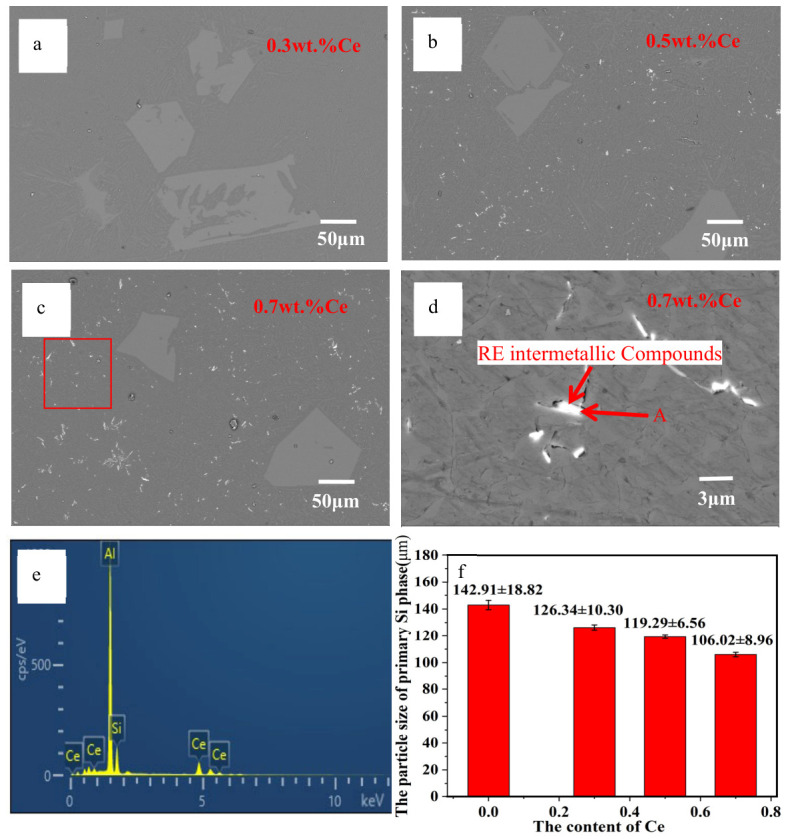
SEM micrographs of Al–20Si–Ce alloy microstructures with different Ce additions: (**a**) 0.3 wt.% Ce; (**b**) 0.5 wt.% Ce; (**c**) 0.7 wt.% Ce; (**d**) enlarged image of Figure 3c; (**e**) EDS spectrum of white RE intermetallic compound indicated by point A; and (**f**) the particle size of the primary Si phase under various Ce additions.

Figure 3 exhibits SEM images of Al–20Si–Ce alloy microstructures with different cerium concentrations. Relative to the unmodified Al–20Si alloy, the morphology of primary silicon changes from complex shapes to a more uniform plate-like structure, with an average size reduction from 142.91 ± 18.82 μm in the unmodified specimen to 106.02 ± 8.96 μm in the 0.7 wt.% Ce concentration, as seen in Figure 3a–c,f. The modification mechanism of RE elements on primary silicon in Al–20Si alloys can be explained by the constitutional undercooling theory proposed by Shi et al. [38]. During the solidification process, RE solute atoms are rejected at the solid–liquid interface of primary silicon, which results in the generation of a solute-enriched layer that lowers the local melting point. Constitutional undercooling occurs near this interface when the actual temperature gradient (G) declines below a critical value, destabilizing the silicon growth front and encouraging branching or refinement of the silicon phases. Due to the extremely low solubility of RE elements in silicon, most RE atoms are expelled into the adjacent melt, which produces an RE-rich layer. These adsorbed RE atoms impede the diffusion of silicon atoms to the growth front, thereby promoting the refinement of primary silicon.

A magnified image of Figure 3c indicates white blocky phases embedded within the aluminum matrix (Figure 3d). EDS analysis proves that these phases consist of aluminum, silicon, and cerium (Figure 3e), showing the generating of cerium-containing intermetallic compounds. According to reference [39], the chemical reaction between Ce and the Al–Si melt can be expressed by Equation (1):

Ce + 2Al + 2Si → (Al_2_Si_2_)Ce (1)

Consequently, the white phase is identified as the intermetallic compound Al_2_Si_2_Ce. These cerium-rich intermetallics predominantly nucleate around the primary silicon rather than within it, which further supports the constitutional undercooling mechanism.

### 3.3. Element Distribution in the Alloy

**Figure 4:**
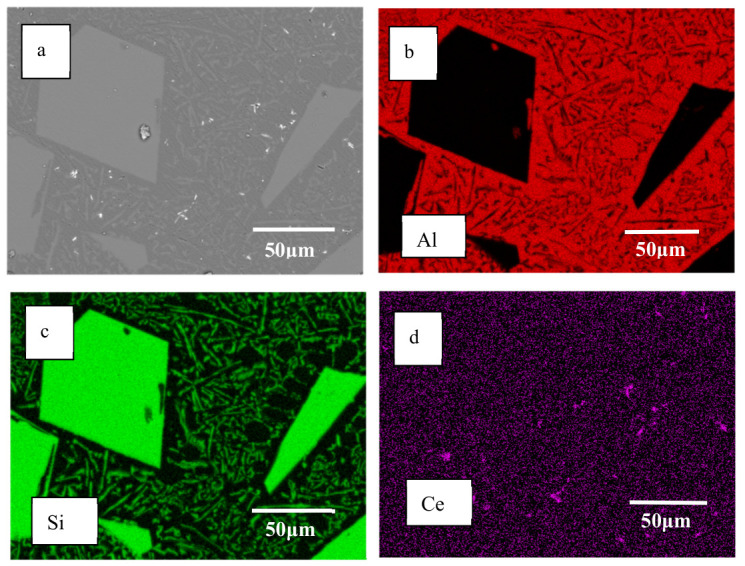
Element distribution images of the Al–20Si–0.7Ce alloy microstructure: (**a**) backscattered electron image, magnification of 500 times; (**b**) distribution of aluminum element; (**c**) distribution of silicon element; and (**d**) distribution of cerium element.

Figure 4 shows the element distribution images of the microstructure for the Al–20Si–0.7Ce alloy. It can be seen from Figure 4a–d that the spatial distributions of the Al and Si elements indicate distinct phase separations, which effectively distinguishes the Al matrix, primary Si, and eutectic Si phases. On the contrary, Ce is primarily dissolved in the Al matrix, with only trace amounts detected in the primary Si phase. This limited presence of Ce in primary Si is because of the low solubility of RE elements in this phase, which leads to a relatively uniform distribution of Ce throughout the Al matrix.

The uniform dispersion of Ce in the Al matrix can be ascribed to three main factors:

(1) High chemical reactivity of Ce: Cerium possesses a strong affinity for impurities such as oxygen, sulfur, and hydrogen, promoting the generation of high-melting-point intermetallic compounds that are uniformly distributed throughout the melt. These intermetallic phases are heterogeneous nucleation sites, which decreases impurity segregation, thereby indirectly promoting an even distribution of Ce.
(2) The melt homogenization effect: the surface tension of the Al–Si melt is decreased in the case of Ce, improving its fluidity and enabling the uniform diffusion of Ce and other alloying elements.
(3) Solidification behavior of Ce: Cerium refines the microstructure of the alloy by decreasing the elemental diffusion distances and reducing the probability of macrosegregation during the solidification process.

In short, the uniform distribution of Ce in the Al matrix aids in modifying the primary Si phase, which in turn helps to enhance the mechanical properties of the alloy.

### 3.4. XRD Analysis

**Figure 5:**
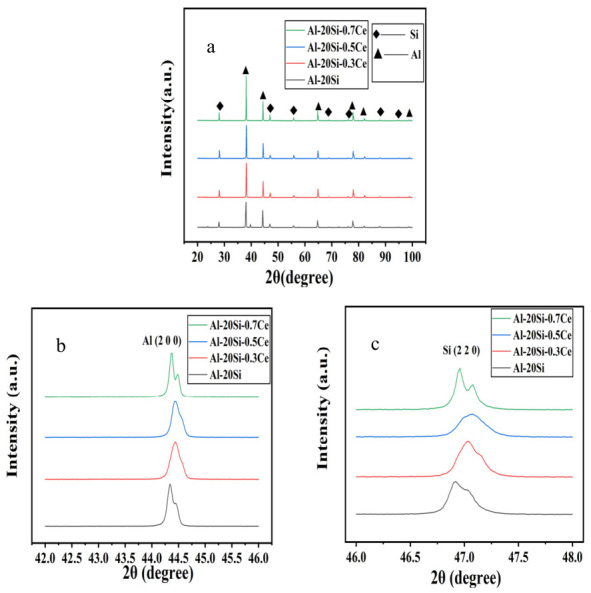
XRD patterns of Al–20Si and Al–20Si–Ce alloys: (**a**) full XRD pattern; (**b**) magnified images of the Al(200) peak; (**c**) magnified images of the Si(220) peak.

Figure 5 indicates the XRD patterns for the Al–20Si and Al–20Si-Ce alloys. As Figure 5a illustrates, the alloys are mainly composed of Al and Si phases. Intermetallic compounds containing cerium were not observed in the XRD patterns, most probably because their concentration is below the detection threshold of XRD (~5 wt.%). Nevertheless, SEM images prove the existence of these phases (Figure 3d).

It is worth noting that the expanded XRD spectra indicate that the diffraction peaks of Al(200) and Si(220) in the Ce-modified alloy shift to higher diffraction angles (Figure 5b,c). Because the atomic radius of cerium (~181.8 pm [40]) exceeds those of aluminum (~143.1 pm [41]) and silicon (~117.2 pm [41]), the incorporation of Ce into the Al matrix or Si phase results in localized lattice distortions. Cerium has very limited solubility in Al or Si because of the significant atomic size mismatch, and any excess cerium reacts with other elements to generate nanoscale segregations or secondary phases. These phases generate compressive stresses on the matrix lattice, which leads to a reduction in interplanar spacing (d-spacing). According to Bragg’s law, a decrease in d-spacing results in an increase in the diffraction angle (θ), which explains the observed peak shifts toward higher angles in Figure 5b,c. Additionally, the broadening of the Si peaks in Figure 5c shows a significant refinement of the Si phase because of Ce addition, a modification that helps to increase the mechanical properties of the alloy.

### 3.5. TEM Observation

**Figure 6:**
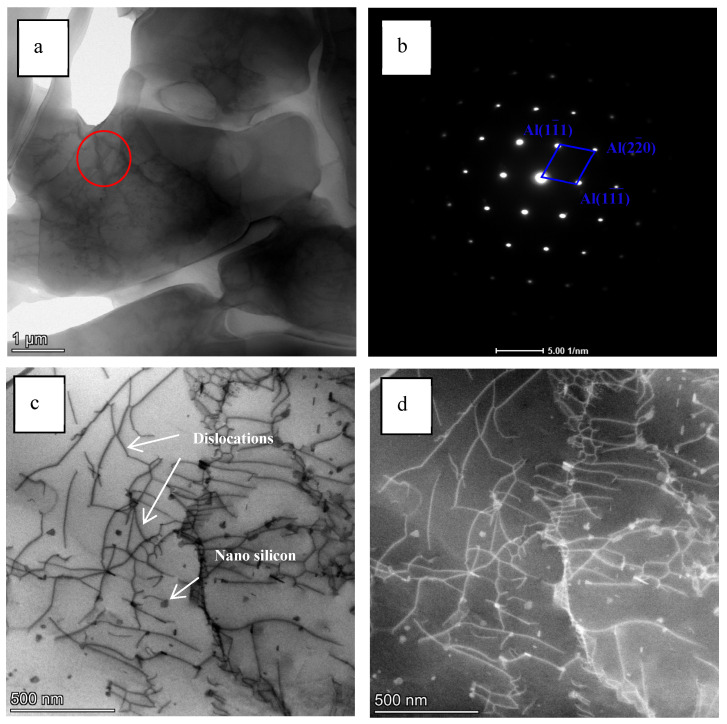
TEM image of the aluminum matrix of the Al–20Si–0.7Ce alloy under STEM mode: (**a**) aluminum matrix; (**b**) SAED pattern corresponding to the aluminum matrix in Figure 6a; (**c**) bright-field phase in an enlarged section of Figure 6a; and (**d**) dark-field phase in an enlarged section of Figure 6a.

Figure 6 exhibits a TEM image of the aluminum matrix in the Al–20Si–0.7Ce alloy, captured in scanning transmission electron microscopy (STEM) mode. It can be seen from Figure 6a that the aluminum matrix consists of numerous grains, with sizes ranging from approximately 500–1200 nm. Figure 6b shows a selected area electron diffraction (SAED) pattern that presents a single-crystal diffraction pattern for the aluminum matrix, which suggests a generally uniform crystallographic orientation among the aluminum grains. It can be seen from Figure 6c,d that further observation indicates the existence of numerous dislocations and dark precipitates in the aluminum matrix. The generation of these dislocations is closely related to the thermal stresses generated during conventional casting processes [42]. When the molten alloy is poured into a metal mold, the surface cools rapidly while the interior cools more slowly, forming a temperature gradient. This uneven cooling causes asynchronous shrinkage between the surface and core, which leads to significant thermal stresses. If these stresses surpass the yield strength of the alloy at the given temperature, they are relieved through plastic deformation, which results in dislocation formation. Stress concentrations at the tips or edges of silicon phases intensify this phenomenon. Sharp stress concentrations occur at the tips or edges of hard, brittle primary silicon particles or coarse eutectic silicon. To accommodate the resulting deformation, the surrounding aluminum matrix withstands plastic deformation, which produces numerous dislocations in the matrix. The dark precipitates highlighted in Figure 6c indicate nanoscale silicon phases with particle sizes ranging from 30 to 40 nm. During the casting process, the molten alloy cools rapidly upon contact with the metal mold, which causes the temperature to drop quickly below the liquidus line and results in significant undercooling. This high level of undercooling significantly increases the nucleation rate of the silicon phase while concurrently inhibiting its diffusion and growth, which in turn promotes the generation of fine, dispersed nanoscale silicon particles. In addition, rapid solidification can cause partial dissolution of silicon atoms into the aluminum matrix, which results in a supersaturated solid solution. The subsequent decomposition of this supersaturated solution leads to the precipitation of nanoscale silicon particles in the aluminum matrix during further solidification processes.

**Figure 7:**
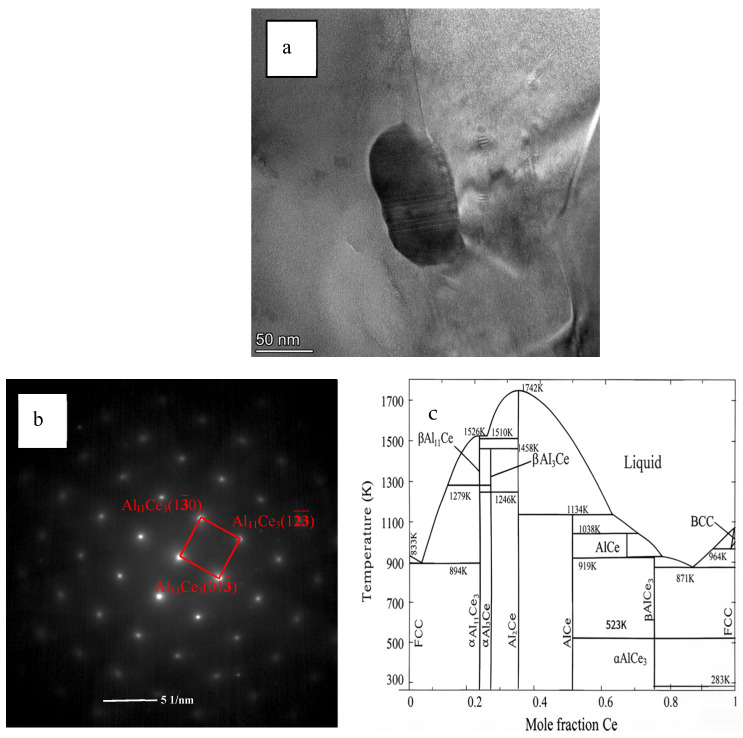
TEM image of the intermetallic α-Al_11_Ce_3_ phase in the Al–20Si–0.7Ce alloy: (**a**) bright-field image of the α-Al_11_Ce_3_ phase; (**b**) SAED pattern corresponding to Figure 7a; and (**c**) Al–Ce binary phase diagram [43].

Figure 7 shows a TEM image of the intermetallic α-Al_11_Ce_3_ phase in the Al–20Si–0.7Ce alloy. As Figure 7a illustrates, the α-Al_11_Ce_3_ phase has an approximately elliptical morphology, with a characteristic dimension of nearly 130 nm. PDF#19-0006 and PDF#48-1841 from the PDF2004 database, analyzed via the crystallographic software Jade6, reveal that the α-Al_11_Ce_3_ phase possesses an orthorhombic crystal structure with a body-centered lattice configuration. Figure 7b shows the corresponding SAED pattern.

According to the Al–Ce binary phase diagram (Figure 7c) [43], the α-Al_11_Ce_3_ phase forms through a eutectic reaction described by the following Equation (2), which occurs in the liquid alloy at 894 K:

L → α-Al + α-Al_11_Ce_3_ (2)

The α-Al_11_Ce_3_ phase is characterized by a fixed stoichiometric atomic ratio of aluminum to cerium atoms at 11:3, a hallmark feature of intermetallic compounds.

**Figure 8:**
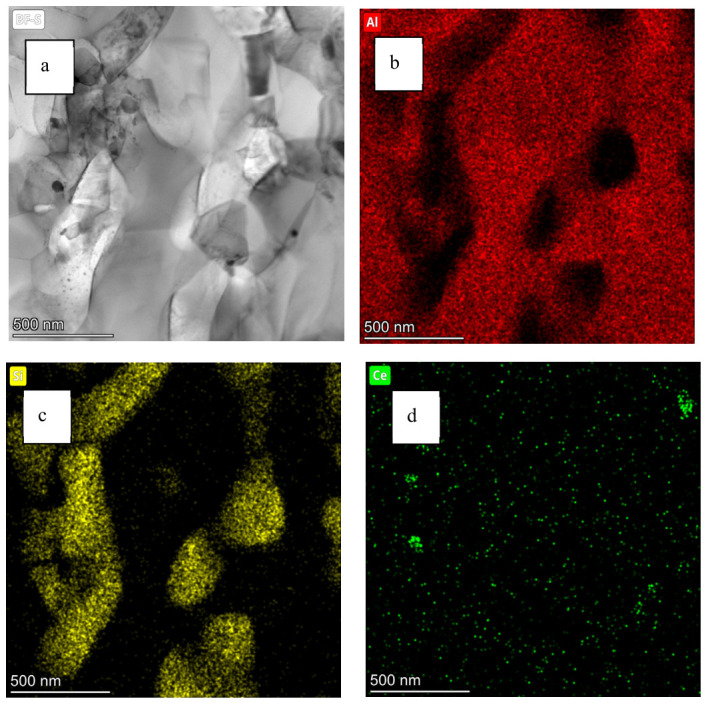
Surface area scan images of the Al–20Si–0.7Ce alloy under the TEM field of view: (**a**) bright-field image; (**b**) distribution of aluminum; (**c**) distribution of silicon; and (**d**) distribution of cerium.

Figure 8 presents elemental mapping images of the Al–20Si–0.7Ce alloy obtained via TEM. In Figure 8a–d, aluminum and silicon exhibit a distinct layered spatial distribution. The silicon phase manifests as eutectic silicon, which presents needle-like and short fibrous morphologies, as seen in Figure 8b,c. Cerium is largely uniformly distributed in the aluminum matrix, with only a small fraction generating the α-Al_11_Ce_3_ intermetallic phase, as seen in Figure 8d. The mechanism behind the uniform dispersion of cerium in the aluminum matrix was previously described in Section 3.3 and is not repeated here. Nevertheless, this homogeneous distribution of cerium plays a crucial role in modifying the primary silicon phase. During the solidification process of the alloy, cerium induces constitutional undercooling, which destabilizes the growth front of the silicon phase, which in turn facilitates branching and refinement. Cerium atoms adsorb at the silicon growth interface, which hinders the diffusion of silicon atoms to this interface, thereby refining the primary silicon morphology. Notably, primary silicon is not visible in Figure 8a, most probably due to its removal during the ion thinning process. In short, aluminum and silicon in the alloy show a distinctly layered distribution, whereas cerium primarily exists in solid solution, with a minor portion generating the α-Al_11_Ce_3_ phase. This element distribution significantly enhances the mechanical properties of the alloy.

### 3.6. Analysis of Mechanical Properties and Fracture Morphology Under Tensile Conditions

**Figure 9:**
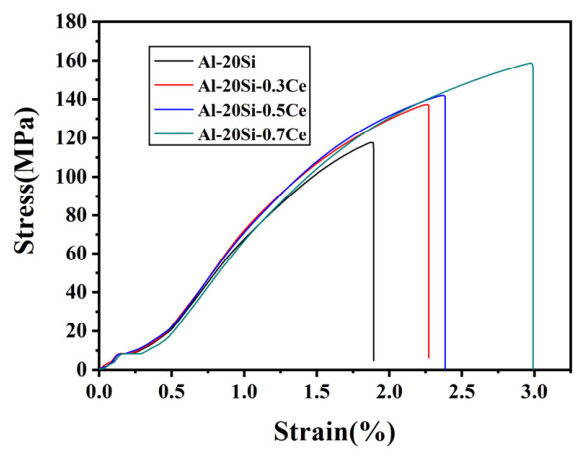
True stress–strain curves of Al–20Si alloys with different Ce additions.

Figure 9 exhibits the true stress–strain curves of Al–20Si alloys with different cerium content. The figure clearly indicates that the flow stress evolution during the deformation process is significantly affected by the addition of Ce. Initially, as deformation begins, the flow stress rapidly increases to a peak value, mainly because of the multiplication and interaction of dislocations, where work hardening is dominant over dynamic softening mechanisms. As strain progresses, dynamic recovery and recrystallization processes, as well as the fragmentation of Si particles, continuously enhance the effects of dynamic softening. When reaching the critical strain, a balance is achieved between work hardening and dynamic softening, which leads to a dynamic equilibrium stage characterized by a constant flow stress despite further straining, indicative of steady-state rheological behavior. Necking starts at the UTS, which results in localized cross-sectional reduction and an increase of true stress. Once the work hardening capacity is exhausted, deformation becomes localized in the necking region until fracture occurs. The refinement of primary Si particles hinder crack initiation because of Ce modification, which thereby enhances tensile strength. Consequently, the UTS of Al–20Si alloys increases with Ce addition, which achieves a maximum value of 153 ± 7 MPa at 0.7 wt.% Ce. Furthermore, the elongation and yield strength data presented in Table 3 indicate that, relative to the unmodified Al–20Si alloy, the elongation of the Ce-modified alloy is enhanced. Specifically, the elongation attains a maximum value of 1.76 ± 0.296% at a Ce concentration of 0.3 wt.%, corresponding to an increase of 53%. At this concentration, the minimum yield strength is measured at 97 ± 1 MPa, reflecting an improvement in the alloy’s plasticity. Beyond this Ce content, tensile strength continues to increase, whereas elongation decreases. Nonetheless, both tensile strength and elongation of the Ce-modified alloy remain superior to those of the unmodified alloy. Previous studies [44,45] have demonstrated that cracks typically initiate at the brittle primary silicon surface. In this context, Li et al. [29] incorporated Er elements to modify the primary silicon in the Al–20% Si alloy, effectively mitigating or preventing premature crack initiation and fracture during tensile testing, thereby substantially enhancing both tensile strength and elongation.

**Figure 10:**
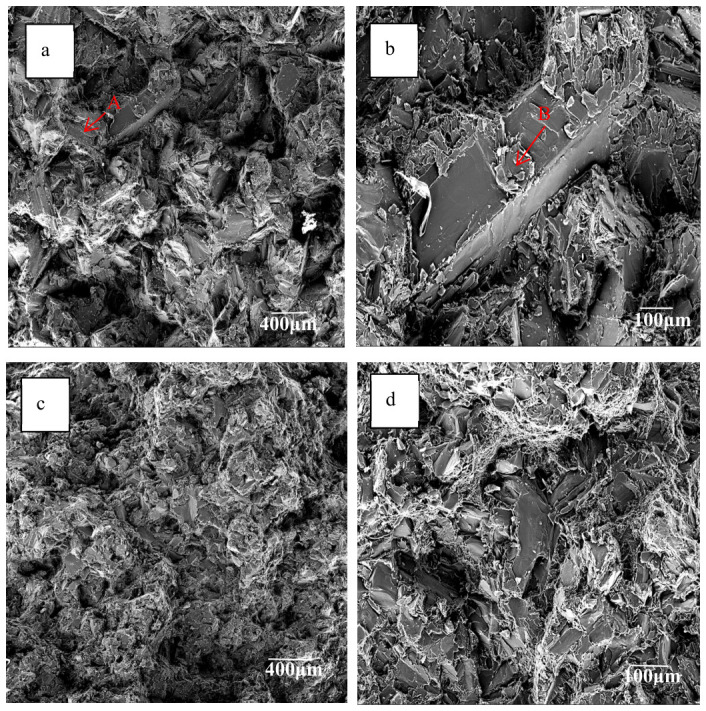
Fracture morphologies of unmodified and Ce-modified Al–20Si alloys under SEM view: (**a**) Al–20Si alloy, low magnification; (**b**) Al–20Si alloy, high magnification; (**c**) Al–20Si–0.7Ce alloy, low magnification; and (**d**) Al–20Si–0.7Ce alloy, high magnification.

Figure 10 exhibits SEM images of the fracture morphologies for both unmodified and Ce-modified Al–20Si alloys. It can be seen from Figure 10a that the fracture surface of the unmodified specimen indicates characteristic cleavage features. The fractured primary Si exhibits flat cleavage facets and river patterns, suggesting crack propagation within the primary Si, as noted at point A in Figure 10a. This proves that cracks initiate and propagate along specific crystallographic planes in the primary Si. In addition, fragmented Si phases appear on the surface of plate-like primary Si, as highlighted at point B in Figure 10b. This fragmentation is ascribed to sharp corners on Si particles and weak interfaces between Si and the aluminum matrix, which generates localized stress concentrations under external loading. When these stress concentrations surpass the critical cleavage fracture strength of Si, microcracks preferentially nucleate along specific crystallographic planes, such as {111}, and rapidly propagate along cleavage planes in the Si phase. Internal micro-defects, including microvoids or inclusions related to coarse primary Si, do not stop crack propagation but may induce crack branching. The complex stress state in the particles can also promote simultaneous crack nucleation on multiple cleavage planes, thus forming intersecting and interconnected cracks and leading to partial fragmentation of the primary Si under complex loading conditions. On the contrary, it can be seen from Figure 10d that no fragmentation of the primary Si is observed after Ce addition, showing that the addition of Ce mitigates stress concentration effects. Moreover, a lot of dimples near the primary Si in Figure 10a,c indicate plastic deformation in the aluminum matrix. These dimples act as energy absorption sites during the fracture process, which promotes plastic deformation. Table 3 demonstrates that the elongation of the alloy containing added cerium surpasses that of the unmodified alloy, indicating a marked enhancement in the material’s plasticity and a fundamental alteration in its fracture mechanism. In short, relative to the unmodified Al–20Si alloy, the Ce-modified alloy shows a significant reduction in primary Si particle size, fewer fractured primary Si particles, a more uniform distribution of dimples, and a reduced area of cleavage fracture. These observations jointly reveal a shift in the fracture mechanism from cleavage fracture to ductile fracture.

### 3.7. Microhardness Analysis

**Figure 11:**
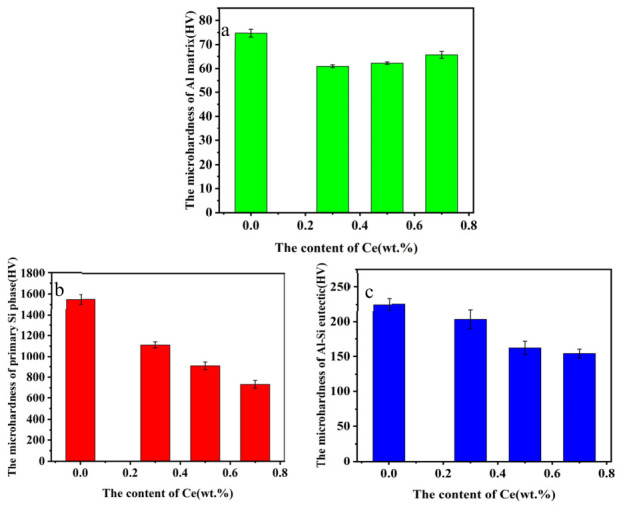
Microhardness results of Al–20Si and Al–20Si–Ce alloys: (**a**) aluminum matrix; (**b**) primary silicon phase; and (**c**) Al–Si eutectic.

Figure 11 exhibits the microhardness results for Al–20Si and Al–20Si–Ce alloys. It can be seen from Figure 11a–c that the microhardness of the aluminum matrix first declines from 75 ± 4 to 61 ± 1 HV, then slightly increases to 62 ± 1 HV with increasing Ce content. The hardness of the primary silicon significantly decreases from 1548 ± 103 to 733 ± 84 HV, and the hardness of the Al–Si eutectic phase also declines from 225 ± 19 to 154 ± 14 HV. This behavior is because of the competing effects of Ce-induced toughening and solid solution strengthening. The generation of soft Al–Ce phases in the alloys indicates the toughening effect of Ce, declining the hardness of both the aluminum matrix and eutectic structure. Meanwhile, the diffusion of Ce and Al into the primary silicon contributes to the decrease in hardness. It is worth noting that the hardness value of the aluminum matrix exhibits a modest increase at a Ce concentration of 0.7 wt.%, which shows enhanced solid solution strengthening from greater Ce incorporation. These opposing effects lead to a slight whole increase in the hardness of the aluminum matrix.

Combined with the results of Section 3.6 and Section 3.7, an apparent paradox is found: although the addition of Ce increases the tensile strength of the alloys, it concurrently reduces their surface hardness, which shows an inverse relationship between these properties. This phenomenon is ascribed to microstructure heterogeneity, which is characterized by soft Al–Ce phases of surface coexisting with a strengthened bulk material. The soft Al–Ce phases promote easier deformation under indentation, thereby declining surface hardness. On the contrary, Ce refines the primary silicon phase of the alloy, which transforms its complex morphology into a more uniform lamellar structure. This refinement relieves stress concentration in the primary silicon, delaying crack initiation. Furthermore, Ce facilitates a more uniform distribution of the silicon phase, improving the whole load-bearing capacity of the alloy and increasing tensile strength. The tensile strength indicates a material’s ability to undergo uniform deformation across its bulk is critical, whereas hardness measures resistance to localized plastic deformation. Essentially, hardness and tensile strength represent material properties at different scales: hardness is largely affected by surface microstructures, while tensile strength depends on the bulk microstructure.

## 4. Conclusions

The effects of cerium on the microstructure evolution of Al–20Si alloys were investigated in this study, and the main conclusions are as follows:

1. The as-cast Al–20Si alloy is composed of an aluminum matrix, Al–Si eutectic, and primary silicon phases. The size of the primary silicon is significantly refined because of Ce addition. In the alloy, cerium is found in the forms of Al_2_Si_2_Ce intermetallic compounds, Ce-containing solid solutions, and the α-Al_11_Ce_3_ phase.
2. The characteristics of phases reveals that the Ce-modified alloy primarily contains aluminum and silicon phases. It is important to note that all diffraction peaks for Al and Si shift toward higher angles, and the Si phase shows peak broadening.
3. The cerium addition significantly improves the tensile strength of the Al–20Si alloy at room temperature, relative to the unmodified alloy. The greatest tensile strength of 153 ± 7 MPa is achieved at a cerium concentration of 0.7 wt.%, while the corresponding elongation of the mechanical specimen is 1.36 ± 0.287.
4. The fracture mechanism shifts from cleavage fracture in the unmodified alloy to ductile fracture after Ce addition.
5. The cerium addition reduces the hardness of the aluminum matrix, primary silicon, and eutectic phases relative to the unmodified alloy. The decrease of hardness in the aluminum matrix and eutectic phases is ascribed to the balance between Ce-induced toughening and solid solution strengthening effects, as well as the generation of the relatively soft α-Al_11_Ce_3_ phase and oxide layers. This reduction of the hardness for primary silicon phase is attributed to the diffusion of Ce atoms into its internal structure.

## Figures and Tables

**Table 1:** A summary of studies investigating the effects of RE elements on the mechanical properties in hypereutectic aluminum–silicon alloys.

No.	Alloy Type	Tensile Strength (MPa)		Imp.	Elongation (%)		Imp.	Ref.
		Unmodified	Modified		Unmodified	Modified		
1	Al–20Si–0.5Er	91.5	157.8	72.5%	0.49	1.72	72%	[29]
2	Al–20Si–0.3La	151	167	10.6%	0.80	1.60	100%	[18]
3	Al–20Si–0.4Nd	152.3	234.8	54.2%	0.70	1.40	100%	[30]
4	Al–20Si–0.8Y	94	139	47.9%	–	–	–	[31]
5	Al–20Si–0.6Sm	103	153	48.5%	0.45	0.76	68.8%	[32]
6	Al–20Si–0.5Yb	93	153	64.5%	0.41	0.71	73.6%	[33]
7	Al–16Si–0.8Eu	123	143	16.3%	1.68	4.48	116%	[34]
8	Al–20Si–0.7Ce	127 ± 9	153 ± 7	20.5%	1.15 ± 0.005	1.36 ± 0.287	18.3%	This work

**Table 2:** Nominal chemical compositions of the alloys.

Number	Alloy	Si (wt.%)	Ce (wt.%)	Al (wt.%)
1	Al–20Si	20	/	Bal.
2	Al–20Si–0.3Ce	20	0.3	Bal.
3	Al–20Si–0.5Ce	20	0.5	Bal.
4	Al–20Si–0.7Ce	20	0.7	Bal.

**Table 3:** The data of mechanical properties for Al–20Si and Al–20Si–Ce alloys.

Alloy Type	Tensile Strength (MPa)	Elongation (%)	Yield Strength (MPa)
Al–20Si	127 ± 9	1.15 ± 0.005	103 ± 6
Al–20Si–0.3Ce	134 ± 5	1.76 ± 0.296	97 ± 1
Al–20Si–0.5Ce	137 ± 7	1.56 ± 0.107	103 ± 6
Al–20Si–0.7Ce	153 ± 7	1.36 ± 0.287	108 ± 2

## Data Availability

The original contributions presented in this study are included in the article. Further inquiries can be directed to the corresponding author.
